# The Aquatic Environment as a Reservoir of *Vibrio cholerae* O1 in Hydrographic Basins of the State of Pernambuco, Brazil

**DOI:** 10.1155/2013/746254

**Published:** 2013-02-25

**Authors:** Carina Lucena Mendes-Marques, Vladimir da Mota Silveira Filho, Ana Paula Rocha da Costa, Mariana de Lira Nunes, Sandoval Vieira da Silva Filho, Ângela Cristina Torres de Araújo Figueirôa, Ernesto Hofer, Alzira Maria Paiva de Almeida, Nilma Cintra Leal

**Affiliations:** ^1^Centro de Pesquisas Aggeu Magalhães, FIOCRUZ-PE, Avendia Professor Moraes Rego, s/n. Cidade Universitária, 50670-420 Recife, PE, Brazil; ^2^Laboratório Central de Saúde Pública Dr. Milton Bezerra Sobral (LACEN-PE), Secretaria Estadual de Saúde de Pernambuco (SES-PE), Praça Oswaldo Cruz, s/n. Boa Vista, 50050-911 Recife, PE, Brazil; ^3^Instituto Oswaldo Cruz (IOC), FIOCRUZ-RJ, Avenida Brasil, 4365 Manguinhos, 21040-360 Rio de Janeiro, RJ, Brazil

## Abstract

After the worldwide cholera epidemic in 1993, permanent environmental monitoring of hydrographic basins was established in Pernambuco, Brazil, where cholera is endemic. After a quiescent period, 4 *rfb*N (serogroup O1) positive water samples that were culture negative were detected by multiplex single-tube nested PCR (MSTNPCR); 2 of these were also *ctx*A (cholera toxin) positive. From May to June 2012, 30 *V. cholerae* O1 isolates were obtained by culturing samples. These isolates were analyzed for the presence of virulence genes by PCR, intergenic spacer region 16S-23S PCR (ISR-PCR), and pulsed field gel electrophoresis (PFGE). The isolates were positive for the *rfb*N gene and negative for the assessed pathogenic genes and were classified into 2 groups by ISR and the same profile by PFGE. Close genetic similarity was observed between them (2012) and environmental strains from 2004 to 2005, indicating the permanence of endemic *V. cholerae* O1 in the region.

## 1. Introduction

*Vibrio cholerae* has played a prominent role in human history and has caused several epidemics that caused many deaths worldwide. While more than 200 O serogroups of *V. cholerae* have been identified, only the O1 and O139 serogroups have been associated with epidemics [1]. Other serogroups commonly known as non-O1/non-O139 [2] coexist in the environment with O1 strains [3]. In Brazil, the O1 serogroup has been recognized as the causative agent of past epidemics, but it is likely that other serogroups were involved in recent small outbreaks [4, 5].

Since the last world cholera epidemic in 1993 [6], the Pernambuco (PE) state Department of Health (Secretaria Estadual de Saúde (SES/PE)) has established permanent environmental monitoring of the hydrographic basins in the state for the detection of *V. cholerae* [7]. This investigation was first based on the standard culture procedures of water samples, and later a molecular technique, multiplex single-tube nested PCR (MSTNPCR), is used to target the *ctx*A gene, which encodes cholera toxin subunit A, and the *rfb*N gene, which is specific to serogroup O1 [8]. In May 2012, four *rfb*N positive but not culturable water samples, of which two were *ctx*A (cholera toxin) positive, were detected by MSTNPCR. After that, the monitoring was intensified and, from May to June 2012, 30 *V. cholerae* O1-positive cultures were unexpectedly isolated from four hydrographic basins.

## 2. Methods

### 2.1. Environmental Water Sample Collection and Bacteriological Analysis

The Moore swab technique [9] was used to collect environmental water samples from hydrographic basins, and the samples were submitted to enrichment in alkaline peptone water (APW). The samples were streaked on thiosulfate-citrate-bile-sucrose agar (TCBS) plates and incubated overnight at 37°C. Yellow colonies were tested for the presence of oxidase with oxidase strips and inoculated in Instituto Adolfo Lutz (IAL)/Rugai tube [10] for motility, glucose and lactose fermentation, gas and H_2_S production, phenylalanine, urea, indol, and lysine tests. The O serogroups were determined by a slide agglutination test with polyvalent O1 and O139 and monospecific Inaba, and Ogawa antisera [11].

### 2.2. Molecular Analysis

The isolates were analyzed for the presence of *V. cholerae* virulence genes (*ctx*A, *ctx*B,* tcp*A, *ace*, and *zot*), the serogroup O1-specific gene (*rfb*N), by intergenic spacer region 16S–23S (ISR-PCR) PCR amplification, and pulsed-field gel electrophoresis (PFGE). *Vibrio cholerae* O1 ATCC 569B was used as control. 

### 2.3. DNA Extraction and PCR Reactions

The genomic DNA was extracted by a heat soak DNA extraction procedure based on Keim et al. [12]. One colony grown on BHI plates was suspended in 200 *μ*L of TE 10 : 1 (10 mM Tris-HCl pH 8.0, 1 mM EDTA), boiled for 20 minutes, and immediately used in PCR reactions with primers designed for the amplification of *ctx*A and *tcp*A [13], *zot* and *ace *[14], *ctx*B [15], and *rfb*N [16]. Each PCR reaction consisted of 50 mM KCl, 10 mM Tris-HCl, 2.5 mM MgCl_2_, 400 mM each dNTP, 20 pmol primers, 1 U of *Taq* DNA polymerase (Promega), heat soak DNA sample (10 *μ*L), and sterile water in a final volume of 25 *μ*L. ISR-PCR was performed as previously described [17].

The PCR reactions were conducted in a Biometra T-3000 Genetic Analyzer thermal cycler using standard procedures. The products were electrophoresed in 1% agarose gels containing SYBR Safe DNA gel stain (Invitrogen) and photographed with Kodak 1D Image Analysis software, version 3.5 (Digital Kodak Science).

### 2.4. PFGE

The typing of *V. cholerae* isolates was performed by PFGE according to a PulseNet [18] standardized protocol. *Not*I-HF (New England Biolabs) digested DNA fragments were separated in a CHEF-DR III Bio-Rad system (Contour-Clamped Homogeneous Electric Fields/Bio-Rad, Hercules, CA, USA) in 1% SeaKem Gold agarose (Lonza, Rockland, ME, USA) gels in 0.5% TBE running buffer at 14°C, with a ramping time of 4.5 V/cm for 22 h. The comparisons in the study included one non-O1 isolate (Vc479-1), which was obtained by coproculture from a diarrheic patient in 2012, *V. cholerae* O1 569B ATCC, and three O1 isolates from previous cholera outbreaks in Brazil (Vc460/04, Vc461/04, and Vc499/05). The Lambda PFGE marker (New England Biolabs, Country RD Ipswich, MA, USA) was the molecular weight standard. The ethidium bromide (1 *µ*g/mL) stained bands were visualized under UV light, and images were captured by 1D Image Analysis Software, version 3.5 (Kodak Digital Science, New Haven, CT, USA).

The PFGE profiles were analyzed by visual inspection with NTSYSpc software version 2.11X [19]. The similarity among the samples was determined by Dice's similarity coefficient [20], and a dendrogram was constructed with UPGMA, following a profile cluster analysis as described by Tenover et al. [21].

## 3. Results

### 3.1. PCR

**Figure 1:**
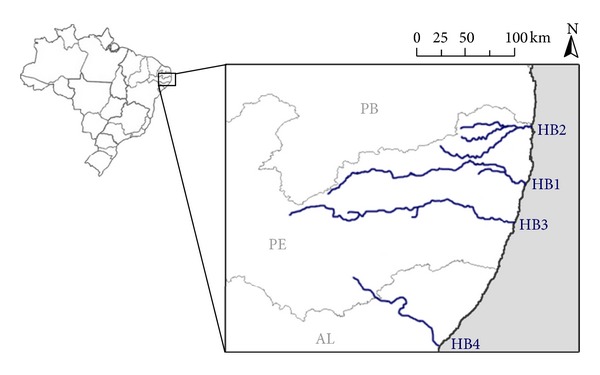
Map of the four hydrographic basins from which *Vibrio cholerae* O1 was isolated. HB1: Capibaribe hydrographic basin, HB2: Goiana hydrographic basin, HB3: Ipojuca hydrographic basin, HB4: Mundaú hydrographic basin, PE: Pernambuco state, PB: Paraíba state; AL: Alagoas state.

MSTNPCR analysis of water samples from aquatic basins of PE, Brazil, from May to June 2012 resulted in the detection of four *rfb*N-positive samples, of which two were also *ctx*A positive. The cultures of these samples were negative. Later on, from May to June 2012, 30 *V. cholerae* isolates were obtained by culture from four hydrographic basins (Figure 1) and classified as O1 by a slide agglutination test. All samples were positive for the *rfb*N gene characterizing the O1 serogroup, and none harbored the pathogenic genes evaluated by PCR.

According to the ISR-PCR patterns generated, the 30 environmental *V. cholerae* O1 isolates were clustered into two distinct groups, named A and B. Group A included all isolates, with the exception of the two isolates (1880 and 1906) that belonged to group B.

### 3.2. PFGE

PFGE analysis of the 30 environmental *V. cholerae* O1 isolates, the non-O1 isolate from a diarrheic patient, the strains of *V. cholerae* O1 569B ATCC, and the previously isolated Brazilian strains Vc460/04, Vc461/04, and Vc499/05 grouped the 35 isolates into six clonally related pulsotypes (A–F).

The 30 *V. cholerae* O1 2012 environmental isolates were shown to be genetically identical, with a 0-1 band difference, and clustered into one pulsotype (A) that was subdivided in two (A1-A2) types. The three Brazilian *V. cholerae* O1 strains from 2004 (Vc460/04, Vc461/04) to 2005 (Vc499/05) were classified into three pulsotypes (B–D). The human isolate (non-O1, Vc479-1) formed a singleton (F), and the reference O1 strain Vc569B was the other singleton (E).

**Figure 2:**
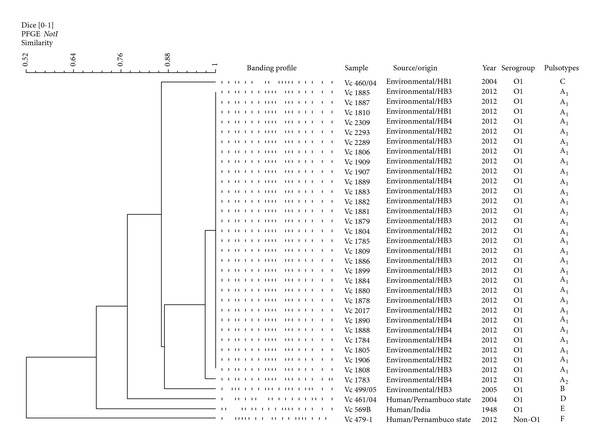
Dendrogram generated by Dice/UPGMA analysis (NTSYS v.2.11X, Applied Biostatistics) of PFGE *Not*I profiles of *Vibrio cholerae* isolates. HB1: Capibaribe hydrographic basin, HB2: Goiana hydrographic basin, HB3: Ipojuca hydrographic basin, HB4: Mundaú hydrographic basin.

A close genetic relationship (~85% similarity) was observed among pulsotype A (2012 Brazilian environmental isolates) and pulsotypes B (a 2005 Brazilian environmental strain—Vc499/05) and C (a 2004 Brazilian environmental strain—Vc460/04), with differences in 4-5 bands (Figure 2).

## 4. Discussion

Through continuous surveillance for cholera in the hydrographic basins of PE, Brazil, four *rfb*N-positive samples, of which two were also *ctx*A positive, were uncovered by MSTNPCR. Although no cultures were isolated, this result suggests the presence of toxigenic *V. cholerae* strains and/or free phage in the aquatic environment of the region.

As recommended [22], this finding triggered prompt control measures by the SES/PE to halt further cholera outbreaks in the infected area. The measures included health education, drinking water treatment (the population was instructed to filter and treat drinking water with sodium hypochlorite, and to cook food thoroughly), and a compulsory search for *V. cholerae* in the environment and in cases of acute diarrhea. Thirty *V. cholerae* strains were further isolated by culture. They were classified as O1 by a slide agglutination test and were positive for the *rfb*N gene characterizing the O1 serogroup, but none harbored the pathogenic genes evaluated by PCR.

These results, together with the presence of nontoxigenic isolates in the same environment, are problematic because phage can infect *V. cholerae* O1 nontoxigenic isolates by the same way it infects other serogroups [5].

The close similarity among the isolates from the 2012, 2005, and 2004 events reveals the permanence of endemic *V. cholerae* O1 in the region. The slight divergences in their PFGE profiles are likely due to accumulated mutations resulting from the adaptive process of the bacteria to environmental and seasonal conditions. As anticipated, the Vc479-1 isolate from a diarrheic patient, although originated from the same place and time period as the cluster A samples, was genetically distinct (≥ differences 7) from the latter as it was revealed to be a non-O1 serogroup isolate. One O1 human 2004 isolate (Vc461/04) and the reference O1 strain (Vc569B) displayed unique pulsotypes and were isolated as singletons, most likely due to their specific toxigenic contents and different geographic origins.

In our study, isolates from different hydrographic basins displayed a clonal profile, possibly due to the presence of cyanobacteria, which are widespread in the region (PE State Department of Health, oral communication). According to Reidl and Klose [23], there is a direct relationship between cholera and algal blooms. It is likely that cyanobacteria are a contributing factor in the permanence of *V. cholerae* O1 in the aquatic environment, which depends on fecal contamination and/or environmental bacterial carriers [24].

In this paper, we report a sudden increase in *V. cholerae* O1 isolation in PE hydrographic basins from May to June 2012. The detection by MSTNPCR of four *rfb*N (serogroup O1)-positive culture-negative water samples, of which two were *ctx*A (cholera toxin) positive, suggests the presence of toxigenic *V. cholerae* strains and/or free phage in the environment. It is likely that phage infects toxigenic and nontoxigenic environmental strains by the same horizontal gene transfer mechanism by which it infects other serogroups. A genetic relationship was identified among *V. cholerae* O1 isolates. These findings indicate the possible emergence of pathogenic strains and the need for permanent monitoring of bacteria in the environment to avoid cholera outbreaks.
